# Prior Contraceptive Use Among Women Who Gave Birth in the US-Mexico Border Region, 2005: The Brownsville-Matamoros Sister City Project for Women’s Health

**Published:** 2008-09-15

**Authors:** Kayan L Lewis, Jose L Robles, Suzanne G Folger, Jill A McDonald, Mirna Perez, Lauren Zapata, Polly A Marchbanks, Mauro Ruiz, Ginger Gossman, Brian C Castrucci, Imelda Garcia

**Affiliations:** Family Health Research and Program Development Unit, Office of Title V and Family Health; Secretariat of Health, Jurisdiction III, Matamoros, Tamaulipas, Mexico; Division of Reproductive Health, National Center for Chronic Disease Prevention and Health Promotion, CDC; Division of Reproductive Health, National Center for Chronic Disease Prevention and Health Promotion, CDC; Division of Reproductive Health, National Center for Chronic Disease Prevention and Health Promotion, CDC; Division of Reproductive Health, National Center for Chronic Disease Prevention and Health Promotion, CDC; Division of Reproductive Health, National Center for Chronic Disease Prevention and Health Promotion, CDC; Public Health Improvement Program, Region 11, Texas Department of State Health Services; Family Health Research and Program Development Unit, Office of Title V and Family Health, Texas Department of State Health Services; Family Health Research and Program Development Unit, Office of Title V and Family Health, Texas Department of State Health Services; Community Health Services, Texas Department of State Health Services

## Abstract

**Introduction:**

Dramatic population growth in  the US-Mexico border region suggests more effective family planning services are needed, yet binational data are scarce. The Brownsville-Matamoros Sister City Project for Women's Health collected binational, standardized data from 947 postpartum women in Cameron County (Texas) and Matamoros (Tamaulipas, Mexico) hospitals from August through November 2005.

**Methods:**

We analyzed these data to estimate the proportion of women with unintended pregnancy and the proportion of these women who reported contraceptive use, and to identify associated factors.

**Results:**

The current pregnancy was unintended for 48% of women overall. Almost half of these women reportedly used birth control at conception, but many used low-efficacy methods. Among women with unintended pregnancy who did not use contraception, 34.1% of Mexico residents believed they could not become pregnant and 28.4% of US residents reported no reason for nonuse. Overall, contraceptive use to prevent pregnancy was less common among younger than older women and among women who had not graduated high school compared with those who had. Among Mexico residents, those who had a source of routine health care were more likely than those who did not to have used contraception.

**Conclusion:**

More effective contraceptive practices are needed in this population, especially among younger and less-educated women. A cooperative binational approach that integrates reproductive and family planning services may be most effective.

## Introduction

Maternal and child health along the border of the United States and Mexico has been identified as an area of concern in need of binational investigation (1,2). The border region is characterized by high birth rates, poverty (relative to the United States), lack of basic services, and dramatic industrial and population growth (3). The border population has proportionately more children and young adults than does the United States overall, and a greater proportion of reproductive-age adults than the overall population of Mexico (4). Given this profile, the need for effective family planning and reproductive health services in the border region is apparent. The impact of such services reaches beyond prevention of unintended pregnancy. Contraceptives provide many health benefits, including protection against sexually transmitted infections (condom), ovarian cysts, and ovarian and endometrial cancers (combined oral contraceptive [COC] pill) and reduction of menstrual pain (COC pill) and blood loss (COC pill and levonorgestrel intrauterine device [IUD]) (5). These are also issues for the border region, which fares poorly compared with the interior of the United States and Mexico for some chronic diseases and sexually transmitted infections (6,7). Other issues unique to the US-Mexico border area are detailed elsewhere in this issue (8).

The risk and consequences of unintended pregnancy are among the most challenging public health issues for border communities. Enhanced family planning services are needed in Mexico (9,10) and in the United States, which has one of the highest adolescent and unintended pregnancy rates among developed countries (11). Little has been published concerning unintended pregnancy and contraceptive practices specific to border residents, however. To our knowledge, the most recent population-based binational survey on contraceptive use among reproductive-age women along the border was conducted in 1979 (12). Major findings of the survey, which was restricted to women who were married (US and Mexican) or in "consensual union" (Mexican), included a higher prevalence of contraceptive use in the United States, at 75% and 65% for non-Hispanic and Hispanic women, respectively, compared with women in Mexico, of whom 50% were using contraception (13). Among US women, 11% of non-Hispanic women and 16% of Hispanic women reported their most recent live birth was unwanted (14); comparable data were not collected for Mexico. A more recent survey in Mexico found that 50% of women with recent births had not planned the pregnancy (15). US data from 2002 indicate that 72.5% of married non-Hispanic women and 59.1% of married Hispanic women were current contraceptive users (16), rates not dramatically different from the 1979 estimates. The 2002 data also showed that 17% of recent live births among Hispanic women and 11% among white women were unwanted; again, these percentages differ little from the 1979 data. A higher prevalence of unintended pregnancy would be expected had unmarried women been included in either study.

The Brownsville-Matamoros Sister City Project for Women's Health (BMSCP) used standardized methods for reproductive health surveillance in 1 pair of sister cities on the US-Mexico border: Brownsville, Texas and Matamoros, Tamaulipas, Mexico (Figure). Surveillance data consisted of in-hospital personal interviews of women after they delivered a live infant. This surveillance pilot project was reviewed for human subjects concerns by the Centers for Disease Control and Prevention (CDC) and was determined to be "nonresearch" or public health practice. Therefore, institutional review board approval was not required. We analyzed BMSCP data to examine the reproductive and contraceptive history of this sample, and to estimate the proportion of women who had not been trying to become pregnant and for whom the pregnancy was unintended. Among women for whom the pregnancy was unintended, we compared those who reported using birth control with those who had not to identify factors associated with attempted prevention of pregnancy.

**Figure 1.**
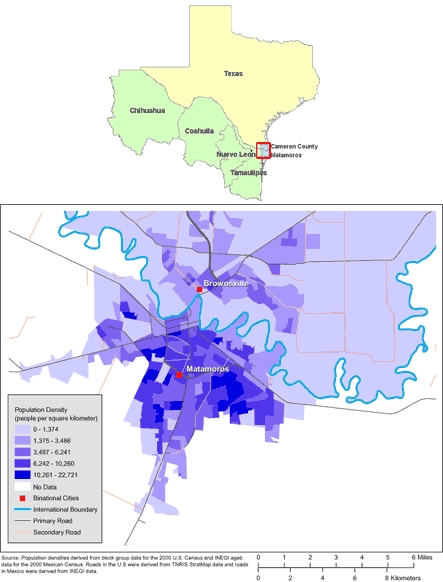
Maps of the US-Mexico Border Region (Top) and of Brownsville, Texas, and Matamoros, Tamaulipas, Mexico (Bottom). (The authors thank Allison Abell Banicki of the Office of Border Health, Texas Department of State Health Services, for creating the map of the Texas-Mexico border states and thank Jean W. Parcher, Sylvia N. Wilson, and the United States Geological Survey [USGS] for providing the map of population density in Brownsville and Matamoros.)

## Methods

The study sample consisted of women who delivered live infants in any of 10 larger hospitals (ie, hospitals with a minimum of 100 deliveries per year) in Matamoros, Tamaulipas, and Cameron County, Texas, from August 21 through November 9, 2005. Women were selected through systematic sampling, stratified by hospital; 947 of 999 (94.8%) sampled women participated. Interviews were conducted in person before hospital discharge. Most interview questions were adapted from surveys used previously in the United States or Mexico; some modifications were necessary to reference the pregnancy leading to the current birth, hereafter referred to as the index pregnancy. More details about BMSCP, including information on site selection, sample design, and response rates, are reported in this issue of *Preventing Chronic Disease* (8).

Our primary outcome of interest was reported birth control use to prevent the (unintended) index pregnancy. The first question about pregnancy intention was, "Thinking back to just before you got pregnant with your new baby, how did you feel about becoming pregnant?" Response options included wanting pregnancy sooner, wanting pregnancy later, wanting pregnancy then, and not wanting pregnancy then or at any time in the future. Pregnancy intention was classified as intended for women who wanted pregnancy sooner or at that time and as unintended for women who wanted pregnancy later or not at all. All women were next asked, "When you got pregnant with your new baby, were you trying to become pregnant?" Women who responded no or "don't know/not sure" were asked, "When you got pregnant with your new baby, were you or your husband or partner doing anything to keep from getting pregnant?" Women who answered no to the question were classified as not having used birth control to prevent the index pregnancy and were asked their reasons for nonuse. Women who answered yes to the question were asked, "When you got pregnant with your new baby, what were you or your husband or partner doing to keep from getting pregnant?" Women who responded by reporting the method(s) of birth control that they and/or their husband or partner had used were classified as having used birth control to prevent the index pregnancy.

Other contraceptive practices examined included ever use of birth control, type and effectiveness of methods first used and used at conception of index pregnancy, and reasons for nonuse among women with unintended index pregnancy who did not use birth control. We used failure rates associated with typical use to classify efficacy of contraceptive methods (5). Methods for which 10% or more of women experience pregnancy during the first year of typical use were classified as less effective; remaining methods were classified as more effective. Less effective methods reported in BMSCP were condoms, spermicides, withdrawal, and rhythm; more effective methods reported were vasectomy, contraceptive pills/patches/injection, and IUD. Several reasons for nonuse were listed in the questionnaire; other reasons reported were entered as text by interviewers. We reviewed text entries and created categories for the following responses: "no reason/don't know," "breastfeeding," and "forgot to use — was not being careful."

Other characteristics examined included demographic factors (country of residence, location of delivery hospital for index pregnancy, country of birth, ethnicity, age, marital status, years of education completed, employment status during 3 months before pregnancy), gravidity (including index pregnancy), cigarette smoking and alcohol consumption in the 2 years before the index pregnancy, and measures of health care access (health care/insurance coverage and source of routine health care). Categories for most variables came directly from questionnaire response options. Categories for education, age, and routine health care source were determined a priori by a binational committee composed of BMSCP partner institution representatives.

We used SUDAAN Release 9.01 (RTI International, Research Triangle Park, North Carolina) for analyses to account for the study sampling design; analyses were conducted for the total sample and separately by country of residence. Weighted percentages and 95% confidence intervals (CIs) were computed to examine the distribution of characteristics of interest. To identify factors potentially associated with contraceptive use at onset of index pregnancy, we computed the weighted percentage of women with such use and 95% CIs according to selected demographic, reproductive, behavioral, and health care access variables. Variables for which χ^2^ tests of independence between categories generated *P* values <.20 and for which cell sizes were not less than 5 were considered of potential importance. These variables were included in the logistic regression model to generate prevalence odds ratios and 95% CIs as measures of association with birth control use at conception of index pregnancy. Variables that appeared relevant for residents of the United States or Mexico were also applied to the model for the other group when applicable. The combined sample model included a variable denoting country of residence. When comparison of logistic regression results for both Mexico and the United States suggested interaction between residency and other factors, interaction terms were modeled. Interaction terms with *P* values <.05 were considered significant.

## Results

Delivery hospital of index pregnancy and medical facility usually used were located in the country of residence for most women (Table 1). Mexico residents were younger and completed fewer years of education than US residents. All Mexico residents and 88.9% of US residents were of Hispanic or Latino ethnicity. Half of the women were married. The proportion of women reporting insurance or health care coverage during pregnancy was similar between US and Mexico residents.

Index pregnancy was unintended for approximately 48% of women overall (Table 2); 66.2% of these unintended pregnancies were mistimed ("wanted later"). Among women who had not been trying to become pregnant or were unsure of pregnancy intention, 46.6% reported that they had been using birth control, and a greater proportion of US (63.4%) than Mexican (48.0%) residents reported use of more effective methods. Reasons for nonuse of birth control were similar between US and Mexico residents. However, a higher percentage of Mexico residents (34.1%) than US residents (13.7%) did not think they could get pregnant, and a higher percentage of women in the United States (28.4%) than in Mexico (3.4%) had no reason or did not know why they didn't use birth control.

Ever use of birth control and use at first sexual intercourse were more common among residents of the United States than of Mexico (Table 2). The contraceptive method first used was one of the less effective methods for 75.6% of women overall.

Among all women who were not trying to get pregnant and had an unintended pregnancy, the proportion who reported birth control use at conception was higher among those who were at least 20 years old, high school graduates, married, and able to access routine health care (Table 3). Additional significant factors detected in bivariate analyses pertained only to US or Mexico residents. Among US residents, women without health care coverage or insurance before pregnancy were more likely to have used contraception than those with such coverage (Table 3). Among Mexico residents, women with at least 1 prior pregnancy were more likely than women with no prior pregnancies to have used contraception.

Results of multivariable logistic regression for the total sample indicated that women younger than 20 were less likely than older women and women with less than an eighth-grade education were less likely than high school graduates to have used birth control  (Table 4). Lower odds of birth control use among US residents and women with no prior births and higher odds of use among women with a private routine health care source were also indicated by the combined model. However, interaction was detected between area of residence and gravidity (*P* value for interaction ≤.001) and between area of residence and routine health care source (*P* value for interaction = .02), indicating the association with area of residence differed across strata for these variables. In country-specific models, a significant effect of gravidity was detected only among residents of Mexico, with a lower likelihood of contraception use among women with no prior births relative to those with 3 or more births (Table 4). Similarly, an increased likelihood of contraception use associated with a private routine health care source was detected only among Mexico residents. When we examined the interaction in the combined model, we found that US residents were more likely than Mexico residents to have used contraception in a first pregnancy but less likely in later pregnancies (first pregnancy prevalence odds ratio [POR], 2.7, 95% CI, 0.9-8.7; second pregnancy POR, 0.2, 95% CI, 0.1-0.7; third or later pregnancy POR, 0.3, 95% CI, 0.2-0.7). Among women with no routine health care source, US residents were less likely than Mexico residents to have used contraception (POR, 0.2; 95% CI, 0.1-0.5); no such difference was detected in contraceptive use between US and Mexico residents with public or private health care.

## Discussion

This study documents a high proportion of unintended pregnancies and provides evidence that enhanced family planning services are needed on both sides of the US-Mexico border. Among women who had just given birth, the pregnancy was unintended for nearly half. Our findings indicate lower odds of birth control use for pregnancy prevention among adolescents and young adult women and women who were not high school graduates. These findings are consistent with other reports (17,18) and indicate demographic groups that may benefit from more directed family planning programs. We also found differences between women living on opposite sides of the border, indicating a need for family planning services to direct efforts toward women in Mexico before their first pregnancy. Other studies have suggested that young women in Mexico may not use effective contraception until after their first child is born (19,20) and that Mexican women of low parity have less consistent contraceptive use (21). Our data do not include measures of consistency or correctness of contraceptive use, but they do indicate a need among Matamoros residents for enhanced family planning services that target nulligravid women and a need for efforts to increase use of effective contraception among young women and women with less education in both areas.

### Access to and effective use of contraception

Overall, residents of the United States and Mexico who had a source for routine health care were more likely than those without routine care to have used birth control to prevent pregnancy, although this difference was significant only for residents of Mexico in the country-specific models. A recent study in Morales, Mexico (22), reported that adolescents without access to Instituto Mexicano del Seguro Social (Mexican Social Security) and Instituto de Seguridad Social Para los Trabajadores del Estado (Social Security System for State Employees in Mexico), both of which are included in our category for health plan or private source of routine health care, were more likely to have an unwanted pregnancy. This finding is not inconsistent with our findings, although unintended pregnancies among the women in our study who used contraception underscore an opportunity for providers of routine health care to promote more effective use of contraception. Our combined model revealed that, among women from both countries who had no source for routine care, US residents were significantly less likely to have used contraception, indicating a need for outreach targeting this group of US residents for family planning services. Additional research would help determine whether the differences within and between countries relate to differences in the ease of obtaining birth control, types of birth control used, or other differences between women with different sources for routine health care.

A substantial number of contraceptive users with an unintended pregnancy reported having used a less effective method when they became pregnant; this was more common among Mexico residents. A recent survey of Hispanic and non-Hispanic white women attending either of 2 publicly funded obstetrics/gynecology clinics in Houston, Texas, found ever use of more effective contraceptive methods and consistent use of any method less common among US-born and non-US-born Hispanic women than among non-Hispanic white women (23). Almost 90% of US residents in BMSCP defined themselves as Hispanic or Latino. The potential for misuse of low-efficacy methods is high (5). Our results also indicate, however, that 48% of Mexico residents and 63% of US residents reported having used hormonal contraceptives (pill, patch, injection), IUD/coil, or vasectomy for contraception — high-efficacy methods — at the time they became pregnant. The low probability of failure for these methods (24) suggests the possibility of reporting error, interviewer error, or another source of misclassification of contraceptive methods used.

### Reasons for unprotected intercourse

Two recent analyses of Pregnancy Risk Assessment Monitoring System data reported reasons for unprotected intercourse among women with an unintended pregnancy. Nettleman et al reported that 33% of women believed they could not get pregnant (25). McDonald et al found that 40.2% of Hispanic and 19.8% of non-Hispanic white women reported this reason for nonuse (26). In BMSCP, 34.1% of women who did not use contraception living in Mexico and 13.7% of nonusers living in the United States believed they could not get pregnant. Such lack of awareness about pregnancy risk provides evidence of a need for education on reproductive health and family planning. Nettleman et al also reported that 30% of nonusers did not mind if they got pregnant; this reason for nonuse was reported by 35.7% of Hispanic and 47.6% of non-Hispanic white women in the McDonald study, but only by 18.2% of BMSCP women. An additional 15.2% of BMSCP nonusers had no reason for nonuse. Women with unintended pregnancy who do not mind if they become pregnant may be ambivalent about pregnancy, as discussed by others (25,27). Ambivalence may also be indicated by women who have no reason for nonuse. In a prospective study of nonpregnant women who visited 1 of 2 California urgent care clinics, Schwarz et al found contraceptive use to be less likely, and use of natural family planning methods or withdrawal more likely, among women who were ambivalent about pregnancy than women who were trying to avoid pregnancy (27).

Women with health care or insurance coverage before pregnancy were less likely than those without coverage to have used birth control to prevent the index pregnancy. These results contradict recent evidence that insured women are more likely to use prescription contraception than uninsured women (28), although insurance coverage may not equate with access to or use of contraception among women on the border. Insurance coverage is not needed to acquire birth control in Mexico, and hormonal methods may be obtained without a prescription. A 1996-1997 postpartum survey of women who gave birth in El Paso, Texas, found that women who lived in the United States were more likely to have obtained pills, injectable contraceptives, or the IUD in Mexico, whereas condoms were more often purchased in the United States (29). Such cross-border acquisition of contraceptives indicates the potential value in a binational collaborative approach to family planning in border communities.

### Limitations

Our study has several limitations. Data were collected retrospectively from postpartum women immediately after birth, thereby excluding women who had used contraception successfully and women who had miscarried or aborted. Contraceptive practices of our study group most likely were less consistent, or involved use of less effective methods, than those of similar sexually active women who did not become pregnant. The proportion of pregnancies that were unintended might have been underestimated or overestimated if women's attitudes about pregnancy changed after delivery. Additionally, women on different sides of the border may have interpreted survey questions differently, although focus groups were conducted in both communities as part of questionnaire development (8). All data in our study are based on self-report and are subject to recall bias/social desirability bias and other sources of misclassification error including interviewer recording error. Our results for contraceptive use by country of residence may have been subject to prevarication bias if women living on one side of the border who gave birth in the adjoining sister city reported that they lived in the country in which they gave birth. Another study involving postpartum interviews of women shortly after delivery in a large hospital on the US-Mexico border considered women's reports of where they lived, obtained prenatal care, and worked and, for multiparous women, locations of prior births and current child care, for coding residency; the initial sample was reduced by 58% after these exclusions (29). Although data are available from BMSCP to apply a more restricted classification of residency, the validity of this approach is unknown and warrants additional research.

The major strength of this study is that it is based on standardized data collected from a large number of women who gave birth in 1 pair of US-Mexico border communities, a group for which such data have been lacking. Participation was high among women on both sides of the border, and the sample provided good coverage of births in the area (8). Many of our analyses focused on the women whose pregnancy was unintended and compared those who reportedly tried to prevent the pregnancy by use of contraception with women who did not use contraception. Given the large proportion of BMSCP pregnancies that were unintended, identifying factors associated with attempted, but failed, pregnancy prevention, in addition to characteristics of women who did not use contraception, can be used as a basis for future work in Cameron County and Matamoros, indicating areas in need of research and possible areas for intervention.

### Conclusion

Developing or expanding culturally relevant approaches to increase awareness of reproductive health and effective contraceptive use is needed to help women and couples in Cameron County and Matamoros achieve their family planning goals. Use of more highly effective and longer-lasting contraceptives that are less prone to user failure may be especially beneficial (30). In both areas, our data suggest that efforts should target women who are young, who have not completed high school, and in Matamoros, before first pregnancy. Effective postpartum counseling emphasizing correct and consistent use of contraceptives is also needed. However, prior reports of contraceptive counseling effectiveness have shown mixed results (31-34); the extent to which these results are relevant for the border region is unknown since they were based on US populations only. Therefore, development of appropriate counseling methods specific for the border population should be considered.

Pregnancy intention is not a simple concept and is recognized to include affective, cognitive, cultural, and contextual dimensions (35). Given the poverty of the region, the documented cost-effectiveness of family planning (36,37), and the extent to which residents receive services in both countries (8), a binational approach is likely to be most effective in expanding the reach of family planning services on the border. Some aspects of family planning knowledge and attitudes about childbearing may differ between women living on different sides of the border (38). More research is needed in this area to examine attitudes and knowledge, to assess content validity of survey questions measuring pregnancy intention and reasons for nonuse of contraception, and perhaps to develop new tools for measurement. Unique approaches for intervention allowing for similarities and differences between the resident populations are needed to help women achieve pregnancy, if desired, at a time that is optimal for them. Continued binational collection of standardized surveillance data such as that collected in BMSCP would allow monitoring of trends and provide informative data for public health programs and policy development in the border region.

## Acknowledgments

The BMSCP was funded through CDC's Division of Reproductive Health and the Office of Global Health Promotion at the National Center for Chronic Disease Prevention and Health Promotion, under a Cooperative Agreement with the United States-Mexico Border Health Association, No. U65 CCU 623699-01-2, and through interagency personnel agreements with the University of Texas at Brownsville and Texas Southmost College and the University of Texas-Houston School of Public Health, Brownsville Regional Campus. In-kind project support was provided by CDC's Division of Health Examination Statistics at the National Center for Health Statistics; the Texas Department of State Health Services, Region 11; the Secretariat of Health, Tamaulipas; and the Mexican Institute of Social Security, Tamaulipas.

In addition to contributions from these partners, support from the following local, regional, and national institutions was critical to the project: the National Center for Gender Equity and Reproductive Health, Mexican Health Secretariat; National Center for Epidemiologic Surveillance and Disease Control, Mexican Health Secretariat; National Center for Health Promotion, Mexican Health Secretariat; National Institute of Statistics, Geography and Informatics, Tamaulipas; Civil Registry, Tamaulipas; Institute for Social Security and Services for State Workers, Tamaulipas; Secretariat of Health, Jurisdiction III, Tamaulipas; Texas Department of State Health Services, Region 11 and Office of Border Health; City of Brownsville Department of Public Health; Cameron County Health Department; Valley Baptist Medical Center in Harlingen; Valley Baptist Medical Center in Brownsville; Valley Regional Medical Center; Harlingen Medical Center; Cameron Park Cultural Center; Brownsville Community Health Center; Dr. Alfredo Pumarejo Lafaurie, General Hospital of Matamoros; Mexican Institute of Social Security General Hospital, Zone #13, Matamoros; Dr Manuel F. Rodríguez Brayda Clinical Hospital, Matamoros; Hospital Guadalupe; Matamoros Center of Family Orientation; Medical Center of Surgical Specialties of Matamoros, and the United States-Mexico Border Health Commission.

## Figures and Tables

**Table 1:** Demographic Characteristics of Women Who Gave Birth in the US-Mexico Border Region, Brownsville-Matamoros Sister City Project for Women's Health, 2005

Characteristic	Residents of United States n = 458) Weighted % (95% CI)	Residents of Mexico (n = 489) Weighted % (95% CI)	All Residents Weighted % (95% CI)
**Country of residence^a^**			
United States	ND	ND	45.8 (42.8-48.8)
Mexico	ND	ND	54.2 (51.2-57.2)
**Country of delivery**			
United States	99.8 (98.6-99.96)	5.0 (3.9-6.5)	48.4 (45.5-51.4)
Mexico	0.2 (0.04-1.5)	95.0 (93.6-96.1)	51.6 (48.6-54.5)
**Location of medical facility usually used by participant^b^**			
United States	92.2 (89.3-94.4)	2.4 (1.6-3.8)	43.4 (40.6-46.2)
Mexico	2.5 (1.5-4.2)	96.0 (94.5-97.1)	53.3 (50.6- 56.1)
Both countries	4.5 (3.2-6.3)	1.2 (0.6-2.3)	2.7 (1.9-3.7)
Do not have a usual medical facility	0.9 (0.4-2.1)	0.4 (0.1-1.4)	0.6 (0.3-1.3)
**Age, y^c^**			
<15	0.2 (0.04-1.2)	0.6 (0.3-1.5)	0.4 (0.2-0.9)
15-19	14.5 (11.7-17.7)	18.6 (15.7-21.9)	16.7 (14.7-19.0)
20-24	30.9 (27.5-34.5)	31.5 (27.8-35.6)	31.2 (28.6-34.0)
25-34	44.1 (40.6-47.8)	42.3 (38.8-46.0)	43.2 (40.7-45.7)
35-39	8.6 (6.2-11.8)	5.3 (3.6-7.6)	6.8 (5.3-8.6)
≥40	1.7 (0.9-3.3)	1.6 (0.9-2.8)	1.7 (1.1-2.5)
**Education completed^d^**			
High school diploma	50.7 (45.9-55.4)	17.1 (13.9-20.8)	32.4 (29.1-35.8)
8-12 y (no diploma)	37.2 (32.8-41.9)	50.9 (46.9-54.9)	44.7 (41.6-47.8)
<8 y	12.1 (9.6-15.3)	32.0 (28.6-35.6)	23.0 (20.7-25.4)
**Ethnicity^e^**			
Hispanic/Latino	88.9 (85.5-91.6)	100	95.0 (93.4-96.2)
Not Hispanic/Latino	11.1 (8.4-14.5)	0	5.0 (3.8-6.6)
**Participant's country of birth^f^**			
United States	56.3 (50.9-61.5)	0.4 (0.1-1.3)	25.7 (22.7-29.0)
Mexico	43.7 (38.6-49.1)	99.6 (98.7-99.9)	74.3 (71.0 –77.3)
**Current marital status^g^**			
Married	49.1 (45.3-53.0)	53.3 (49.2-57.3)	51.4 (48.6-54.2)
Unmarried, not living with partner	26.3 (22.7-30.2)	9.4 (7.3-12.0)	31.5 (29.1-34.0)
Unmarried, living with partner	24.6 (21.7-27.6)	37.3 (33.8-41.0)	17.1 (15.0-19.4)
**Health care or insurance coverage during pregnancy^h^**			
No	30.8 (27.6-34.2)	30.6 (27.9-33.4)	30.7 (28.6-32.8)
Yes	69.2 (65.9-72.4)	69.4 (66.6-72.1)	69.4 (67.2-71.4)
**Health care or insurance coverage just before pregnancy^i^**			
No	74.8 (70.5-78.6)	41.9 (38.2-45.6)	56.9 (54.0-59.8)
Yes	25.3 (21.4-29.5)	58.2 (54.4-61.8)	43.1 (40.2-46.0)
**Source of routine health care^j^**			
None	41.7 (36.4-47.2)	15.1 (12.6-18.1)	27.3 (24.3-30.5)
Public facilities	31.1 (27.3-35.1)	28.3 (25.8-31.1)	29.6 (27.4-31.9)
Health plan/private facilities	27.2 (23.2-31.6)	56.5 (52.7-60.3)	43.1 (40.3-46.0)
**Smoked at least 100 cigarettes during past 2 years^k^**			
No	91.9 (89.6-93.7)	95.1 (93.5-96.3)	93.6 (92.3-94.7)
Yes	8.1 (6.4-10.4)	4.9 (3.7-6.5)	6.4 (5.3-7.7)
**Drank any alcoholic drinks during past 2 years^l^**			
No	56.1 (52.3-59.9)	71.4 (68.1-74.4)	64.4 (61.9-66.8)
Yes	43.9 (40.1-47.8)	28.6 (25.6-31.9)	35.6 (33.2-38.1)
**Employment status^m^**			
Employed	47.9 (42.8-53.1)	49.0 (45.3-52.6)	48.5 (45.4-51.6)
Unemployed	10.7 (7.6-15.0)	4.8 (3.4-6.8)	7.5 (5.8-9.8)
Not in labor force	41.3 (35.8-47.1)	46.2 (42.7-49.7)	44.0 (40.8- 47.3)

Abbreviation: CI, confidence interval; ND, not determined.

a Country of residence was defined as the country in which the respondent reported currently living.

b Data are missing for 7 US residents and 4 Mexico residents.

c Data are missing for 1 US resident.

d Data are missing for 6 US residents and 1 Mexico resident.

e Data are missing for 15 US residents.

f Data are missing for 12 US residents and 4 Mexico residents.

g Current marital status was categorized as married, unmarried but living with partner (defined as a live-in significant other or in a consensual union), and unmarried, not living with partner (defined as separated, widowed, or divorced). Data are missing for 6 US residents and 3 Mexico residents.

h Data are missing for 2 US residents and 1 Mexico resident.

i Data are missing for 1 US resident.

j Defined as the type of medical facility the respondent usually uses for routine health care or when she feels sick. The category "none" includes emergency room, urgent care clinic, or pharmacy, and respondents who said they had no source for routine health care. "Public facilities" include public clinic, health center, SSA (Secretary of Health, Mexico), and local or state hospital. "Health plan/private facilities" include private doctor's office, military (Veterans Affairs Hospital, Sedena Marina Hospital), Instituto Mexicano del Seguro Social (Mexican Social Security), and Instituto de Seguridad Social Para los Trabajadores del Estado (Social Security System for State Employees in Mexico). Data are missing for 2 US residents.

k Data are missing for 4 US residents and 3 Mexico residents.

l Data are missing for 3 US residents and 2 Mexico residents.

m Data are missing for 8 US residents and 1 Mexico resident. Category "not in labor force" includes women who reported that they were homemakers or students, or otherwise unable to work.

**Table 2:** Contraceptive and Reproductive Characteristics of Women Who Gave Birth in the US-Mexico Border Region, Brownsville-Matamoros Sister City Project for Women's Health, 2005^a^

Characteristic	Residents of United States (n = 458) Weighted % (95% CI)	Residents of Mexico (n = 489) Weighted % (95% CI)	All Residents Weighted % (95% CI)
**Number of pregnancies (including current pregnancy)^b^**			
≥3	46.6 (41.9-51.4)	34.8 (31.7-38.0)	40.2 (37.4-43.1)
2	23.6 (20.4-27.2)	30.1 (27.7-32.6)	27.1 (25.2-29.2)
1	29.8 (26.1-33.7)	35.1 (31.5-38.9)	32.7 (30.1-35.3)
**Pregnancy intention^c^**			
Pregnancy intended	50.5 (46.7-54.3)	52.7 (49.7-55.8)	51.7 (49.3-54.1)
Pregnancy not intended	49.5 (45.7-53.3)	47.3 (44.2-50.3)	48.3 (45.9-50.7)
**Trying to become pregnant when pregnancy occurred^d^**			
No	57.4 (54.0-60.7)	46.6 (43.4-49.9)	51.5 (49.2-53.8)
Yes	42.4 (39.2-45.7)	53.2 (49.8-56.5)	48.3 (46.0-50.6)
**Use of birth control when became pregnant (this pregnancy)^e^**			
No	56.5 (51.5-61.4)	50.1 (43.9-56.3)	53.4 (49.4- 57.3)
Yes	43.5 (38.6-48.5)	49.9 (43.7-56.1)	46.6 (42.7-50.6)
**If yes to used birth control when became pregnant, all methods of use when became pregnant (this pregnancy)**			
Pill (only)	39.9 (31.1-49.3)	12.0 (7.5-18.6)	25.2 (20.2-30.9)
Condom (only)	27.6 (20.4-36.2)	24.8 (18.9-31.9)	26.1 (21.4-31.5)
Foam/jelly/cream (only)	0.9 (0.2-4.8)	0	0.4 (0.1-2.3)
Injection (only)	12.3 (7.8-18.8)	15.8 (10.6-23.0)	14.1 (10.5-18.9)
Patch (only)	4.4 (2.1-8.8)	0.8 (0.1-4.6)	2.5 (1.3-4.9)
Diaphragm/sponge/cervical cap (only)	0	0	0
IUD/coil (only)	3.5 (1.5-8.1)	19.4 (13.0-27.9)	11.9 (8.2-16.9)
Withdrawal (only)	1.8 (0.5-5.8)	11.4 (7.7-16.6)	6.9 (4.8-9.7)
Rhythm (only)	5.3 (2.9-9.5)	8.8 (5.1-14.5)	7.1 (4.8-10.5)
Emergency contraception (only)	0	0	0
Tubes tied (only)	0	0	0
Vasectomy (only)	0	0	0
Other (specify)	2.6 (1.0-6.9)	0	1.2 (0.5-3.3)
More than 1: condom and other only (eg, vasectomy, injection, breastfeeding infant, rhythm, withdrawal)	1.8 (0.5-5.8)	5.3 (2.4-11.1)	3.6 (1.9-6.8)
More than 1: withdrawal and rhythm only	0	1.8 (0.6-5.6)	0.9 (0.3-3.0)
**Effectiveness of birth control method used when became pregnant^f^**			
More effective with typical use	63.4 (53.4-72.4)	48.0 (40.0-56.1)	55.2 (49.2-61.1)
Less effective with typical use	36.6 (27.6-46.6)	52.0 (43.9-60.0)	44.8 (38.9-50.9)
**Reason for no birth control use among women not trying to get pregnant^g^**			
Didn't mind if got pregnant	17.4 (11.5-25.4)	18.8 (12.4-27.5)	18.2 (13.7- 23.8)
Thought I couldn't get pregnant	13.7 (9.6-19.1)	34.1 (27.6-41.2)	24.5 (20.4-29.1)
Had side effects from using birth control	10.0 (5.8-16.8)	7.2 (4.0-12.6)	8.5 (5.7-12.5)
Problems getting birth control when needed	3.7 (1.5-8.4)	0	1.7 (0.7-4.0)
Thought husband/partner was sterile	1.0 (0.2-5.5)	0.9 (0.2-5.1)	0.9 (0.3-3.2)
Husband/partner didn't want to use anything	6.6 (2.7-15.2)	9.9 (6.4-15.0)	8.4 (5.4-12.7)
No reason/don't know	28.4 (20.3-38.2)	3.4 (1.5-7.7)	15.2 (11.1-20.3)
Breastfeeding	0.9 (0.2-4.9)	6.3 (3.2-12.2)	3.8 (2.0-7.0)
Forgot to use birth control — was not being careful	0	5.2 (2.4-11.2)	2.8 (1.2-6.2)
Other	18.4 (12.2-26.7)	14.1 (9.9-19.8)	16.1 (12.2-20.9)
**Ever use of birth control^h^**			
No	17.8 (14.0-22.3)	35.5 (31.5-39.8)	27.5 (24.6- 30.7)
Yes	82.3 (77.7-86.1)	64.5 (60.2-68.5)	72.5 (69.3-75.4)
**Birth control use during first sexual intercourse^i^**			
No	55.8 (51.4-60.1)	75.3 (70.7-79.5)	66.5 (63.1-69.8)
Yes	44.2 (39.9-48.6)	24.7 (20.6-29.3)	33.5 (30.2-36.9)
**Effectiveness of birth control method first used^j^**			
More effective with typical use	21.7 (16.3-28.3)	28.5 (23.0-34.7)	24.5 (20.3-29.1)
Less effective with typical use	78.3 (71.7-83.7)	71.5 (65.3-77.0)	75.6 (70.9-79.7)

Abbreviations: CI, confidence interval; IUD, intrauterine device.

a Country of residence was defined as the country in which the respondent reported currently living.

b Data are missing for 1 US resident.

c Pregnancy is classified as intended for women who wanted to be pregnant sooner or wanted to be pregnant then, and classified as unintended for women who wanted to be pregnant later or did not want pregnancy then or any time in the future. Eleven (2.4%) Cameron County residents and 2 (0.4%) Matamoros residents reported "don't know" or "not sure" and these were excluded from the dichotomous classification. Data are missing for 17 US residents and 3 Mexico residents.

d Data for 6 US residents and 1 Mexico resident who refused to answer the question and from 1 resident of each country who answered "don't know" are excluded from the percentage estimates.

e Only asked of residents who were not trying to become pregnant or said that they were not sure or didn't know whether they were trying (n = 260 US residents; n = 229 Mexico residents).

f More effective methods with typical use reported included contraceptive pill, patch, injection, IUD/coil, and vasectomy. Less effective methods with typical use reported included contraceptive foam, jelly and cream, condoms, diaphragm, rhythm, and withdrawal. The US had 3 "other" responses that could not be categorized by efficacy.

g 30 nonuser US residents and 3 nonuser Mexico residents did not provide a reason for nonuse but did not report that they had no reason or that they did not know why they did not use, and are excluded from this analysis.

h Data are missing for 18 US residents and 4 Mexico residents.

i Data are missing for 18 US residents and 6 Mexico residents.

j More effective methods with typical use reported included contraceptive pill, patch, injection, IUD/coil, and vasectomy. Less effective methods with typical use reported included contraceptive foam/jelly/cream, condoms, diaphragm, rhythm, and withdrawal. Data were missing for 2 US residents.

**Table 3:** Bivariate Analyses of Women With Unintended Index Pregnancy Who Reported Birth Control Use at Conception, by Selected Characteristics and Country of Residence, Brownsville-Matamoros Sister City Project for Women's Health, 2005

Characteristic	Residents of United States (n = 205) Weighted % (95% CI)	Residents of Mexico (n = 197) Weighted % (95% CI)	All Residents Weighted % (95% CI)
**Country of residence**			
United States	ND	ND	47.3 (41.1- 53.7)
Mexico	ND	ND	54.9 (47.9- 61.7)
**Country of delivery**			
United States	47.6 (41.3-54.0)	53.4 (30.7-74.9)	48.0 (42.1-53.9)
Mexico	0^a^	55.0 (47.7-62.1)	54.7 (47.3-61.9)
**Age, y^b^**			
<20	34.2 (20.3-51.5)	26.2 (16.0-39.7)	29.4 (21.2-39.3)
≥20	50.0 (42.6-57.4)	63.6 (56.1-70.5)	56.8 (51.5-61.9)
**Education completed^c^**			
High school diploma	55.1 (47.3-62.7)	74.7 (57.6-86.5)	59.7 (52.5-66.5)
8-12 y (no diploma)	35.9 (26.0-47.2)	53.4 (42.9-63.5)	46.3 (38.7-54.1)
<8 y	51.5 (34.3-68.4)	49.3 (39.7-59.1)	49.9 (41.3-58.6)
**Ethnicity^d^**			
Hispanic or Latino	49.5 (42.4-56.7)	54.9 (47.9-61.7)	52.5 (47.5-57.4)
Not Hispanic or Latino	35.6 (21.6-52.5)	0	35.6 (21.6-52.5)
**Participant's country of birth**			
United States	45.2 (38.2-52.4)	50.0 (6.3-93.7)^e^	45.3 (38.3– 52.5)
Mexico	48.8 (38.0-59.6)	55.5 (48.1-62.6)	53.5 (47.3-59.6)
**Current marital status^f^**			
Married	53.2 (44.4-61.7)	63.9 (51.3-74.8)	58.9 (51.2-66.1)
Unmarried, not living with partner	46.4 (35.7-57.6)	42.5 (24.4-62.9)	45.3 (36.1-54.9)
Unmarried, living with partner	39.7 (30.3-49.9)	51.2 (41.5-60.9)	47.0 (40.2-54.0)
**Health care coverage just before pregnancy^g^**			
No	49.4 (43.2-55.7)	49.5 (38.7-60.3)	49.5 (43.9-55.0)
Yes	35.2 (22.2-50.8)	59.1 (50.8-66.9)	54.3 (47.3-61.2)
**Source of routine health care^h^^,^ i**			
None	42.9 (32.6-53.9)	42.4 (32.2-53.3)	42.8 (34.9-51.0)
Public facilities	52.5 (40.3-64.3)	45.1 (33.6-57.2)	48.7 (40.2-57.3)
Health plan/private facilities	49.0 (37.1-61.1)	65.9 (54.4-75.8)	60.7 (51.9-68.8)
**Smoked at least 100 cigarettes in past 2 years**			
No	47.6 (41.0-54.3)	56.5 (49.5-63.3)	52.2 (47.5-57.0)
Yes	41.4 (20.4-66.2)	34.0 (13.0-64.0)	38.7 (22.1-58.5)
**Drank any alcoholic drinks during past 2 years**			
No	50.0 (42.9-57.2)	54.3 (45.9-62.4)	52.4 (46.7-58.1)
Yes	43.5 (33.1-54.4)	56.3 (43.9-67.9)	49.0 (41.4-56.6)
**Number of pregnancies (includes current pregnancy)^j^**			
≥3	51.0 (41.6-60.3)	69.1 (61.0-76.1)	59.4 (52.9-65.7)
2	40.1 (29.2-52.1)	62.7 (48.8-74.8)	53.0 (44.0-61.7)
1	46.3 (35.0-58.1)	26.3 (16.3-39.5)	35.3 (27.5-44.1)
**Employment status**			
Employed	49.3 (40.4-58.2)	55.1 (43.9-65.9)	52.3 (45.3-59.2)
Unemployed	52.2 (35.9-68.0)	74.7 (44.2-91.7)	57.7 (42.9-71.2)
Not in labor force^k^	44.7 (34.7-55.1)	53.3 (44.7-61.7)	49.5 (42.8-56.2)

Abbreviations: CI, confidence interval; ND, not determined.

a No US residents delivered in Matamoros.

b Proportion using birth control differs between categories for US residents, *P* = .17, and Mexico residents, *P* <.001.

c Proportion using birth control differs between categories for US residents, *P* = .04, and Mexico residents, *P* = .04.

d All Mexico residents were Hispanic.

e Only 2 Mexico residents were born in the United States.

f Current marital status was categorized as married, unmarried but living with partner (defined as a live-in significant other or in a consensual union), and unmarried, not living with partner (defined as separated, widowed, or divorced). Data are missing for 6 US residents and 3 Mexico residents. Proportion using birth control differs between categories for US residents, *P* = .02, and Mexico residents, *P* = .12.

g Proportion using birth control differs between categories for US residents, *P* = .04, and Mexico residents, *P* = .12.

h Defined as the type of medical facility the respondent usually uses for routine health care or when she feels sick. The category "none" includes emergency room, urgent care clinic, or pharmacy, and respondents who said they had no source for routine health care. "Public facilities" include public clinic, health center, SSA (Secretary of Health, Mexico), and local or state hospital. "Health plan/private facilities" include private doctor's office, military (Veterans Affairs Hospital, Sedena Marina Hospital), Instituto Mexicano del Seguro Social (Mexican Social Security), and Instituto de Seguridad Social Para los Trabajadores del Estado (Social Security System for State Employees in Mexico). Data are missing for 2 US residents.

i Proportion using birth control differs between categories for US residents, *P* = .001.

j Proportion using birth control differs between categories for Mexico residents, *P* <.001.

k Homemaker, student, retired, or otherwise unable to work.

**Table 4:** Factors Associated With Birth Control Use at Conception Among Women With Unintended Pregnancy, Brownsville-Matamoros Sister City Project for Women's Health, 2005

Characteristic	Prevalence Odds Ratios (95% CIs)		

	Residents of United States (n = 205)	Residents of Mexico (n = 195)^a^	Combined Model (N = 400)
**Country of residence**			
Mexico	ND	ND	1.0
United States	ND	ND	0.5 (0.3-0.8)
**Age, y**			
≥20	1.0	1.0	1.0
<20	0.7 (0.3-2.0)	0.6 (0.3-1.1)	0.5 (0.3-0.9)
**Education completed**			
High school diploma	1.0	1.0	1.0
8-12 y (no diploma)	0.5 (0.2-1.0)	0.3 (0.1-1.1)	0.5 (0.3-1.0)
<8 y	0.8 (0.3-1.9)	0.2 (0.1-0.6)	0.5 (0.3-0.9)
**Current marital status^b^**			
Married	1.0	1.0	1.0
Unmarried, not living with partner	1.0 (0.6-1.6)	1.2 (0.5-3.1)	0.9 (0.6-1.5)
Unmarried, living with partner	0.7 (0.4-1.1)	1.0 (0.6-1.9)	0.8 (0.5-1.1)
**Health care coverage before pregnancy**			
No	1.0	1.0	1.0
Yes	0.5 (0.3-0.9)	0.6 (0.3-1.1)	0.7 (0.4-1.0)
**Source of routine health care^c^**			
None	1.0	1.0	1.0
Public facilities	1.7 (0.8-3.8)	1.4 (0.7-2.8)	1.5 (0.9-2.6)
Health plan/private facilities	1.2 (0.6-2.4)	3.4 (1.6-7.1)	2.0 (1.1-3.5)
**Number of pregnancies (includes current pregnancy)**			
≥3	1.0	1.0	1.0
2	0.7 (0.4-1.5)	0.8 (0.3-2.1)	0.8 (0.5-1.4)
1	0.8 (0.4-1.6)	0.1 (0.04-0.3)	0.4 (0.2-0.7)

Abbreviations: CI, confidence interval; ND, not determined.

a Sample is not 197 because of missing data and listwise deletion.

b Current marital status was categorized as married, unmarried but living with partner (defined as a live-in significant other or in a consensual union), and unmarried, not living with partner (defined as separated, widowed, or divorced). Data are missing for 6 US residents and 3 Mexico residents.

c Defined as the type of medical facility the respondent usually uses for routine health care or when she feels sick. The category "none" includes emergency room, urgent care clinic, or pharmacy, and respondents who said they had no source for routine health care. "Public facilities" include public clinic, health center, SSA (Secretary of Health, Mexico), and local or state hospital. "Health plan/private facilities" include private doctor's office, military (Veterans Affairs Hospital, Sedena Marina Hospital), Instituto Mexicano del Seguro Social (Mexican Social Security), and Instituto de Seguridad Social Para los Trabajadores del Estado (Social Security System for State Employees in Mexico).

## References

[1] Nightingale EO, Soberon G, Peck NG, Mortimer AM (1992). En ambos lados: investigación colaborativa binacional para mejorar la atención materno-infantil en la región de la frontera México-Estados Unidos. Border Health.

[2] (2003). United States-Mexico Border Health Commission. Healthy Border 2010: an agenda for improving health on the United States-Mexico Border.

[3] Ruiz-Beltran M, Kamau JK (2001). The socio-economic and cultural impediments to well-being along the US-Mexico border. J Community Health.

[4] Soden DL (2006). At the cross roads: US/Mexico border counties in transition.

[5] Trussell J, Hatcher RA, Trussell J, Shamah-Levy T, Rojas R (2007). Choosing a contraceptive: efficacy, safety, and personal considerations. Contraceptive technology.

[6] Olaiz-Fernandez G, Rivera-Dommarco J, Shamah-Levy T, Rojas R, Rillalpando-Herenandez S, Herenandez-Avila M (2006). Encuesta Nacional de Salud y Nutricion 2006.

[7] Baldwin SB, Djambazov B, Papenfuss M, Abrahamsen M, Denman C, Guernsey de Zapien J (2004). Chlamydial infection in women along the US-Mexico Border. Int J STD AIDS.

[8] McDonald JA, Johnson CH, Smith R, Folger SG, Chavez AL, Mishra N (2008). Reproductive Health Surveillance in the US-Mexico Border Region, 2003-2006: The Brownsville-Matamoros Sister City Project for Women’s Health. Prev Chronic Dis.

[9] Programa sectorial de salud: México. Secretaría de salud 2007-2012.

[10] Programa nacional de salud 2001-2006.

[11] Espey E, Cosgrove E, Ogburn T (2007). Family planning American style: why it's so hard to control birth in the US. Obstet Gynecol Clin North Am.

[12] Smith JC, Warren CW, Garcia Nunez (1983). The U.S.-Mexico border: contraceptive use and maternal health care in perspective. A report of survey information on reproductive age women living in the border areas of the United States and Mexico, 1979.

[13] Warren CW, Smith JC, Garcia-Nunez J, Rochat RW, Martinez-Manautou J (1981). Contraceptive use and family planning services along the U.S.-Mexico border.. Int Fam Plan Perspect.

[14] Rochat RW, Warren CW, Smith JC, Holck SE, Friedman JS (1981). Family planning practices among Anglo and Hispanic women in U.S. counties bordering Mexico. Fam Plann Perspect.

[15] (1993). El entorno de la regulacion de la fecundidad en mexico. Serie: resultados de investigation.

[16] Chandra A, Martinez GM, Mosher WD, Abma JC, Jones J (2005). Fertility, family planning, and reproductive health of U.S. women: data from the 2002 National Survey of Family Growth. Vital Health Stat 23.

[17] (1997). Women of the world: laws and policies affecting their reproductive lives: Latin America and the Caribbean.

[18] Finer LB, Henshaw SK (2006). Disparities in rates of unintended pregnancy in the United States, 1994 and 2001. Perspect Sex Reprod Health.

[19] Barber SL (2007). Family planning advice and postpartum contraceptive use among low-income women in Mexico. Int Fam Plan Perspect.

[20] Atkin LC, Alatorre-Rico J (1992). Pregnant again? Psychosocial predictors of short-interval repeat pregnancy among adolescent mothers in Mexico City. J Adolesc Health.

[21] Langer A, Harper C, Garcia-Barrios C, Schiavon R, Heimburger A, Elul B (1999). Emergency contraception in Mexico City: what do health care providers and potential users know and think about it?. Contraception.

[22] Núñez-Urquiza RM, Hernández-Prado B, García-Barrios C, González D, Walker D (2003). [Unwanted adolescent pregnancy and post-partum utilization of contraceptive methods.]. Salud Publica Mex.

[23] Sangi-Haghpeykar H, Ali N, Posner S, Poindexter AN (2006). Disparities in contraceptive knowledge, attitude and use between Hispanic and non-Hispanic whites. Contraception.

[24] Trussell J (2004). Contraceptive failure in the United States. Contraception.

[25] Nettleman MD, Chung H, Brewer J, Ayoola A, Reed PL (2007). Reasons for unprotected intercourse: analysis of the PRAMS survey. Contraception.

[26] McDonald JA, Suellentrop K, Paulozzi LJ, Morrow B (2008). Reproductive health of the rapidly growing Hispanic population: data from the Pregnancy Risk Assessment Monitoring System, 2002. Matern Child Health J.

[27] Schwarz EB, Lohr PA, Gold MA, Gerbert B (2007). Prevalence and correlates of ambivalence towards pregnancy among nonpregnant women. Contraception.

[28] Culwell KR, Feinglass J (2007). Changes in prescription contraceptive use, 1995-2002: the effect of insurance status. Obstet Gynecol.

[29] Potter JE, Moore AM, Byrd TL (2003). Cross-border procurement of contraception: estimates from a postpartum survey in El Paso, Texas. Contraception.

[30] Stevens-Simon C, Kelly L, Kulick R (2001). A village would be nice but . . . it takes a long-acting contraceptive to prevent repeat adolescent pregnancies. Am J Prev Med.

[31] Lesnewski R (2004). Preventing unintended pregnancy: implications for physicians. Am Fam Physician.

[32] Forrest JD, Frost JJ (1996). The family planning attitudes and experiences of low-income women. Fam Plann Perspect.

[33] Weisman CS, Maccannon DS, Henderson JT, Shortridge E, Orso CL (2002). Contraceptive counseling in managed care: preventing unintended pregnancy in adults. Womens Health Issues.

[34] Moos MK, Bartholomew NE, Lohr KN (2003). Counseling in the clinical setting to prevent unintended pregnancy: an evidence-based research agenda. Contraception.

[35] Santelli J, Rochat R, Hatfield-Timajchy K, Gilbert BC, Curtis K, Cabral R (2003). The measure and meaning of unintended pregnancy. Perspect Sex Reprod Health.

[36] Trussell J, Leveque JA, Koenig JD, London R, Borden S, Henneberry J (1995). The economic value of contraception: a comparison of 15 methods. Am J Public Health.

[37] Trussell J (2007). The cost of unintended pregnancy in the United States. Contraception.

[38] Russell AY, Williams MS, Farr PA, Schwab AJ, Plattsmier S (1993). Patterns of contraceptive use and pregnancy among young Hispanic women on the Texas-Mexico border. J Adolesc Health.

